# Modeling of Gas Production from Shale Reservoirs Considering Multiple Transport Mechanisms

**DOI:** 10.1371/journal.pone.0143649

**Published:** 2015-12-14

**Authors:** Chaohua Guo, Mingzhen Wei, Hong Liu

**Affiliations:** 1 Department of Petroleum Engineering, Missouri University of Science and Technology, Rolla, MO, 65401, United States of America; 2 Department of Petroleum Engineering, Chongqing University of Science and Technology, Chongqing, 401331, China; Tsinghua University, CHINA

## Abstract

Gas transport in unconventional shale strata is a multi-mechanism-coupling process that is different from the process observed in conventional reservoirs. In micro fractures which are inborn or induced by hydraulic stimulation, viscous flow dominates. And gas surface diffusion and gas desorption should be further considered in organic nano pores. Also, the Klinkenberg effect should be considered when dealing with the gas transport problem. In addition, following two factors can play significant roles under certain circumstances but have not received enough attention in previous models. During pressure depletion, gas viscosity will change with Knudsen number; and pore radius will increase when the adsorption gas desorbs from the pore wall. In this paper, a comprehensive mathematical model that incorporates all known mechanisms for simulating gas flow in shale strata is presented. The objective of this study was to provide a more accurate reservoir model for simulation based on the flow mechanisms in the pore scale and formation geometry. Complex mechanisms, including viscous flow, Knudsen diffusion, slip flow, and desorption, are optionally integrated into different continua in the model. Sensitivity analysis was conducted to evaluate the effect of different mechanisms on the gas production. The results showed that adsorption and gas viscosity change will have a great impact on gas production. Ignoring one of following scenarios, such as adsorption, gas permeability change, gas viscosity change, or pore radius change, will underestimate gas production.

## Introduction

Due to the increasing energy shortage in recent years, gas production from shale strata has played an increasingly important role in the volatile energy industry of North America and is gradually becoming a key component in the world’s energy supply [1, 2]. Shale strata or shale gas reservoirs (SGR) are typical unconventional resources with a critically low transport capability in matrix and numerous “natural” fractures. Core experiments have shown that 90% of shale bedrock permeability are less than 150 nd, and diameters of the pore throat are 4~200 nm [3]. Natural gas has been stored in SGRs in three forms [2]: (a) as free gas in the micro fractures and nano pores; (b) as dissolved gas in the kerogen [4]; and (c) as adsorption gas in the surface of the bedrock. About 20%~85% of gas in SGRs is stored in form [5].

Gas transport in extremely low-permeability shale formations is a complex process with many co-existing mechanisms, such as viscous flow, Knudsen diffusion, slip flow [6, 7], and gas adsorption-desorption. Some scholars have studied gas transport behavior in SGRs. E. Ozkan used a dual-mechanism approach to consider transient Darcy and diffusive flows in the matrix and stress-dependent permeability in the fractures for naturally fractured SGRs [8]. However, they ignored the effects of adsorption and desorption. Moridis considered Darcy’s flow, non-Darcy flow, stress-sensitivity, gas slippage, non-isothermal effects, and Langmuir isotherm desorption [9]. They found that the production data from tight-sand reservoirs can be adequately represented without accounting for gas adsorption whereas there will be significant deviations if gas adsorption were omitted in SGRs. But they did not consider gas diffusion in the Korogen. Bustin studied the effect of fabric on gas production from shale strata. However, it was assumed the matrix does not have viscous flow or diffusion mechanisms. It was also assumed that gas transport in the fracture system obeys Darcy’s law, which is not sufficiently accurate [10]. Yu-Shu Wu proposed an improved methodology to simulate shale gas production, but the gas adsorption-desorption were ignored in the model [11]. It is necessary to develop a mathematical model that incorporates all known mechanisms to describe the gas transport behavior in tight shale formations.

The simulation work was greatly simplified when the apparent permeability was first introduced by Javadpour. In 2009, the concept of apparent permeability, considering Knudsen diffusion, slippage flow, and advection flow, was proposed [4]. Through this method, the flux vector term can be simply expressed in the form of Darcy’s equation, which greatly reduces the computation complexity. Then the concept of apparent permeability was further applied to pore scale modeling for shale gas [12, 13]. Civan and Ziarani derived the expression for apparent permeability in the form of the Knudsen number [14, 15] on a unified Hagen-Poiseuille equation [16].

In this paper, a general numerical model for SGRs was proposed which was constructed based on the dual porosity model (DPM). Mass balance equations were constructed for both matrix and fracture systems using the dusty gas model. In the matrix, Knudsen diffusion, gas desorption, and viscous flow were considered. Gas desorption was characterized by the Langmuir isothermal equation. In the fracture, viscous flow and non-darcy slip permeability were considered. The increase of the pore radius due to gas desorption was calculated from the gas desorption volume from the pore wall. Gas viscosity was characterized as a function of the Knudsen number. Weak form equations were derived for the system based on the constant pressure boundary and got solved using COMSOL. A comprehensive sensitivity analysis was conducted, and a detailed investigation was done to determine their impact on the shale gas production performance. The results showed that considering gas viscosity change greatly increased gas production under given reservoir conditions and slowed down the production decline curve. Considering pore radius increase due to gas desorption from the pore wall resulted in a higher production, but the effect was not very significant under the given reservoir conditions. In SGRs, both the matrix and fracture permeability changed during production. Ignoring one of these factors such as Knudsen diffusion, slippage effect, desorption, viscosity decrease, and porosity increase would lead to a lower cumulative production. Therefore, it is crucial to incorporate these factors into the existing models to obtain a more accurate shale gas production prediction.

## Pore Distribution and Gas Transport Process in SGRs

**Fig 1:**
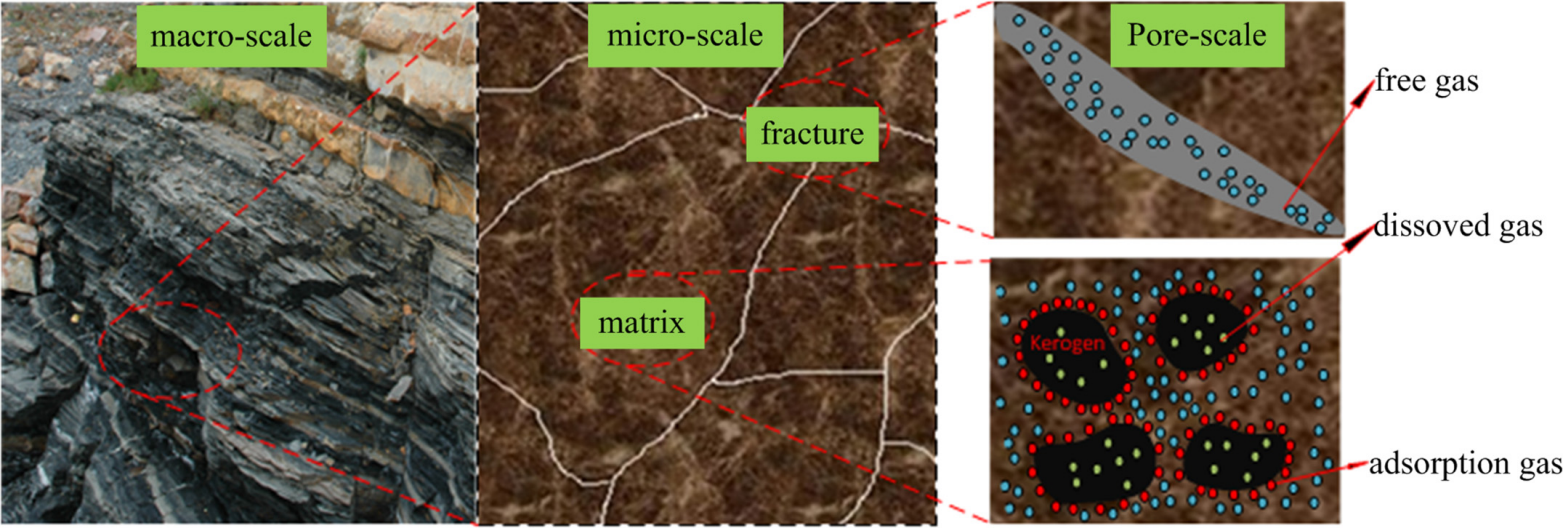
Gas distribution in shale strata from macro-scale to micro-scale. In the fracture there exist free gas and in the matrix free gas and adsorption gas co-exist.

Understanding the pore distribution and geometry of shale strata is of great importance in understanding the gas transport process in the media. Fig 1 shows the gas distribution and geometry of shale strata from micro to macro scales. It informs us that in the fracture, only free gas exists. And in the matrix which is full of kerogen, free gas and adsorption gas co-exist. In such a complicated system, gas desorbs from the pore wall and transports in the matrix system. Due to pressure difference between the matrix and fracture system, gas transfers between the matrix and fracture. Then, gas flows to the wellbore and is produced to the surface [3]. This model constructed in this study was inspired according to this gas transport process in shale strata.

## Model Construction

This theoretical study was based on the following basic assumptions:

1. Single component, one phase flow in SGRs;
2. Ignore the effect of gravity and heterogeneity on the gas flow;
3. Ideal gas behavior for the natural gas in shales, and gas deviation factor z = 1;
4. Formation rocks were incompressible, and the porosity change due to rock deformation was ignored;
5. Isothermal flow process was present in the whole reservoir life;
6. Gas adsorption-desorption kinetics obeyed the Langmuir curve, and can achieve equilibrium state quickly at any reservoir pressure (diffuse quickly).

Consider a generalized mass balance equation [16] in every grid block is as follows:

$$\frac{dM}{dt}+\nabla \cdot (\rho \;\vec{u})=Q$$ (1)

where M is mass accumulation term; **u** is velocity; ρ is density; Q is source term; and t denotes time.

**Fig 2:**
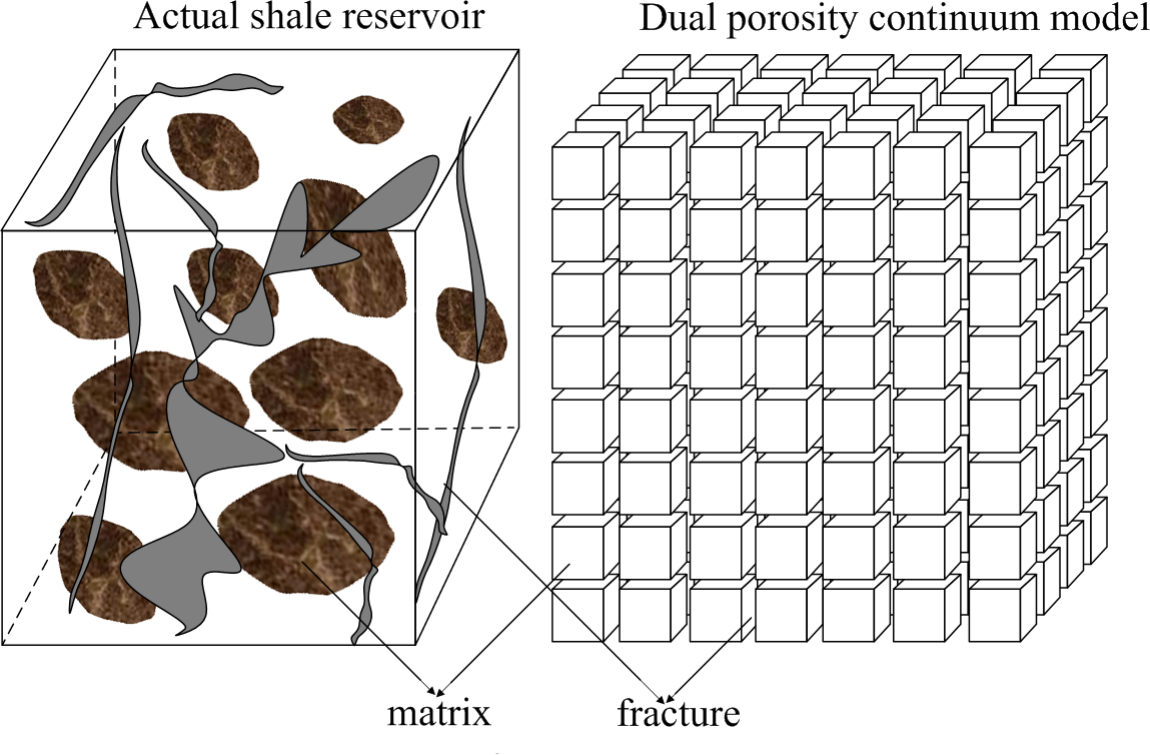
Idealization of the heterogeneous porous medium as DPM.

For shale strata which are full of micro-fractures, two general methods can be used to deal with the fracture-matrix interaction. One is the dual porosity continuum method (DPCM), including dual porosity and dual permeability (DPDM) [17], or MINC (multiple interacting continua) [18]. The other one is the explicit discrete fracture model [19]. Though the later model is more rigorous, it is still limited in applications to field study due to its computational intensity and lack of detailed knowledge of fracture distribution [14]. In this study, the DPCM, which is shown in the Fig 2, was used in this study to describe the complex fracture distribution.

For the DPCM, there are two mass balance equations that correspond to fracture and matrix systems, respectively, as indicated by Warren and Root [17].

With the subscripts *f* and *m* representing fracture and matrix system, respectively, the two sets of equations are illustrated as follows:

$${\left(\frac{dM}{dt}\right)}_{f}+{\left(\nabla \cdot (\rho \;\vec{u})\right)}_{f}=Q_{f}$$ (2)

$${\left(\frac{dM}{dt}\right)}_{m}+{\left(\nabla \cdot (\rho \;\vec{u})\right)}_{m}=Q_{m}$$ (3)

The first term on the left side is the mass accumulation term; the second term on the left side is the flow vector term; and the right side is the source/sink term.These terms are addressed correspondingly in the following sub-sections.

### Mass accumulation term

The general form of the mass accumulation term is:

$$M=\phi \sum_{\beta }\;S_{\beta }\;\rho_{\beta }$$ (4)

where *β* denotes the fluid phase, *ϕ* is porosity, *S*_*β*_ is the faction of pore volume occupied by the phase *β*, *ρ*_*β*_ is the density of the phase *β*. Specifically for a gas reservoir, *S*_*β*_ = 1.

However, in matrix system of shale strata, there are adsorption gas and free gas in the system which is shown in Fig 1. The free gas, which occupies the pores of the matrix, can be represented by M_free_ = ϕ∑_β_S_β_ρ_β_, and the adsorbed gas in the surface of the bulk matrix can be represented as *M*_*adsorp*_ = ∑(1 − *ϕ*)*q*_*a*_, where *q*_*a*_ is the adsorption gas volume per unit bulk volume.

Gas desorption occurs when the pressure difference exists in organic grids (kerogen). With free gas production, the pressure in the pores decreases, which results in the pressure difference between the bulk matrix and the pores. Due to this pressure drop, the gas desorbs from the surface of the bulk matrix. The most commonly used empirical model that describes the sorption and desorption of gas in shale and provides a reasonable fit to most experimental data is the Langmuir single-layer isotherm model [12, 14, 20, 21], which is expressed in Eq (5):

$$V_{ads}=\frac{v_{L}\cdot p}{p_{L}+p}$$ (5)

where v_L_ is the Langmuir volume, denoting the amount of gas sorbed at infinite pressure *p*_∞_, and p_L_ is the Langmuir pressure, corresponding to the pressure at which half of the Langmuir volume v_L_ is reached. Normally, v_L_ and p_L_ are measured under standard temperature and pressure (STP) conditions.

So, the adsorption gas volume per unit of bulk volume can be expressed in Eq (6):

$$q_{a}=\frac{\rho_{s}M_{g}}{V_{std}}V_{std}=\frac{\rho_{s}M_{g}}{V_{std}}\frac{V_{L}p_{m}}{p_{L}+p_{m}}$$ (6)

where q_a_ is the adsorption gas per unit area of the shale surface, kg/m^3^; V_std_ is the mole volume under standard condition (0°C, 1atm), m^3^/mol; q_std_ is the adsorption volume per unit mass of shale, m^3^/kg; V_L_ is the Langmuir volume, m^3^/kg; p_L_ is the Langmuir pressure, *P*_*a*_; and ρ_s_ is the density of shale core, kg/m^3^.

The mass accumulation considering free gas and adsorption gas can be represented as *M* = ∑(*ϕρ*_*β*_ + (1 − *ϕ*)*q*_*a*_), and the corresponding partial differential form is $\frac{d}{dt}\int_{V_{n}}M\;dV_{n}=\frac{\partial (\rho_{g}\phi )}{\partial t}+\frac{\partial [q_{g}(1-\phi )]}{\partial t}$.

Based on the equation of state (EOS): $pV=nRT=\frac{m}{M}RT,\;\rho_{g}=\frac{m}{V}=\frac{pM}{ZRT}=p\gamma$

$$\frac{\partial q_{a}}{\partial t}=\frac{\partial q_{a}}{\partial p_{m}}*\frac{\partial p_{m}}{\partial t}=\frac{\partial }{\partial p_{m}}\left(\frac{\rho_{s}M_{g}}{V_{std}}\frac{V_{L}p_{m}}{p_{L}+p_{m}}\right)*\frac{\partial p_{m}}{\partial t}=\frac{M_{g}p_{L}V_{L}\rho_{s}}{V_{std}{\left(p_{L}+p_{m}\right)}^{2}}\frac{\partial p_{m}}{\partial t}$$ (7)

$${\left(\frac{dM}{dt}\right)}_{m}=\frac{\partial (\rho_{g}\phi )}{\partial t}+\frac{\partial [q_{g}(1-\phi )]}{\partial t}=\left[\gamma \phi_{m}+\frac{(1-\phi_{m})M_{g}p_{L}V_{L}\rho_{s}}{V_{std}{\left(p_{L}+p_{m}\right)}^{2}}\right]\frac{\partial p_{m}}{\partial t}$$ (8)

As there is only free gas in the fracture system, the mass accumulation term can be described as follows:

$${\left(\frac{dM}{dt}\right)}_{f}=\frac{\partial (\rho_{g}\phi ))}{\partial t}=[\gamma \varphi_{f}]\frac{\partial p_{f}}{\partial t}$$ (9)

### Flow vector term

**Fig 3:**
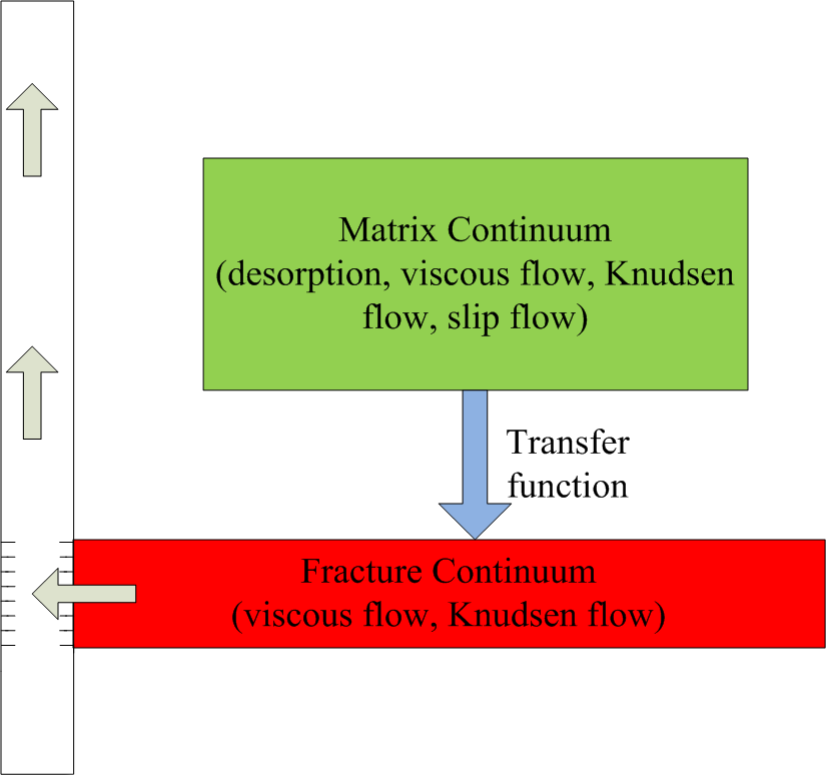
Transport scheme of shale gas production in DPM. Gas desorbed from the matrix surface and transferred to the fracture, then flow into the wellbore. Non-darcy flow, Knudsen diffusion, slip flow, and viscous flow have been considered.

This paper distinguishes the gas flow in shale gas reservoirs by flowing media: matrix and fracture systems. Due to the differences of pore sizes in those two media, gas flow mechanisms are different. Fig 3 shows the general flow process from matrix to fracture, then to wellbore in the shale strata.

### Flow vector term in the matrix

The general Darcy’s law informs us the relationship between velocity and pressure drop, as presented in the Eq (10):

$$\vec{u}=-\frac{1}{\mu }\vec{K}\cdot (\nabla p+\rho g\cdot \nabla z)$$ (10)

**Fig 4:**
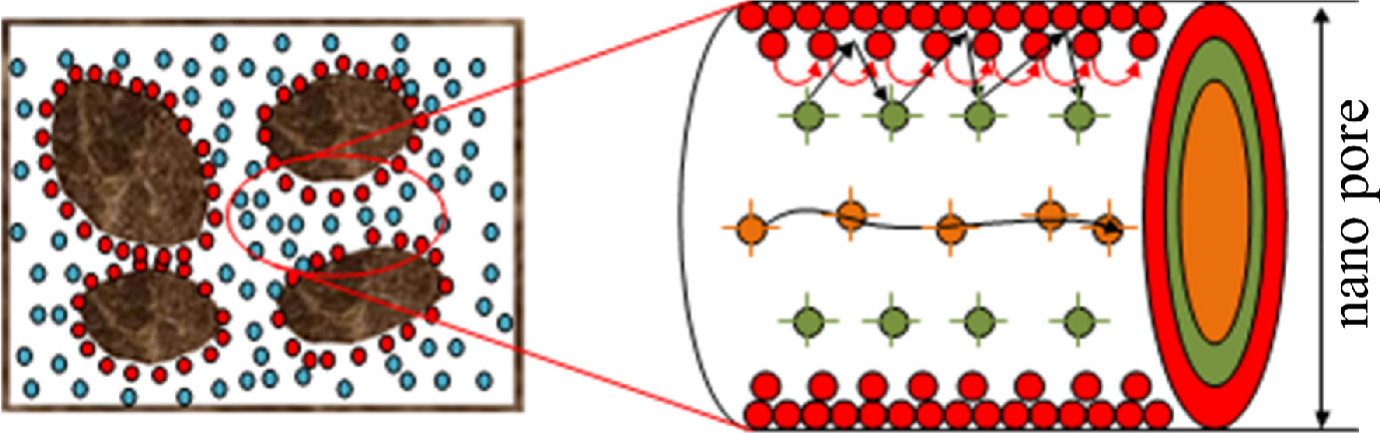
Gas flow mechanisms in a nano pore. Red solid dots represent Knudsen diffusion, while blue ones represent viscous flow.

For low density fluids such as gases, it is assumed that the effect of gravity can be ignored. Therefore, a simplified empirical form of Darcy’s law can be used for the flow vector term in pores ranging from tens to hundreds of microns:

$$\nabla .(\rho \;\vec{u})=-\nabla .\left(\rho (\frac{\vec{K}}{\mu }\nabla p)\right)$$ (11)

where $\vec{K}$ is the permeability tensor. In the matrix system, where pores are in the range of nano-meters, the conventional Darcy’s law cannot be used to describe the flow process. Bird et al. concluded that gas transportation in nano pores is a multi-mechanism-coupling process that includes Knudsen diffusion, viscous convection, and slip flow. Fig 4 shows the flow mechanisms of gas transport in the nano pores of shale strata [22]. The following subsections describe those flow mechanisms further.

#### (1). Viscous flow

When the mean free path of the gas is smaller than the pore diameter (Knudsen number is far less than 1), then the motion of the gas molecules is mainly affected by the collision between molecules. There exists viscous flow caused by the pressure gradient between the single component gas, which can be described by Darcy’s law [23]:

$$J_{v}=U.\nabla C=-\frac{{\rho_{m}k}_{mi}}{\mu_{g}}(\nabla p_{m})$$ (12)

where J_v_ is the mass flow (kg/(m^2^ · s)), ρ_m_ is gas density in bedrock (kg/ m^3^), k_mi_ is the intrinsic permeability (Guo et al., 2015) of bedrock (m^2^), μ_g_ is the gas viscosity (Pa · s), and p_m_ is the pore pressure in the bedrock (Pa).

#### (2). Knudsen Diffusion

When the pore diameter is small enough so that the mean free path of the gas is close to the pore diameter (i.e., Knudsen number > 1), the collision between the gas molecules and the wall surface dominates. The gas mass flow can be expressed by the Knudsen diffusion [23]:

$$J_{k}=-M_{g}D_{km}(\nabla C_{m})$$ (13)

where J_k_ is the mass flow caused by Knudsen diffusion (kg/(m2 · s)), M_g_ is the gas molar mass (kg/mol), D_km_ is the diffusion coefficient of the bedrock (m2/s), and C_m_ is the gas mole concentration (mol/ m^3^).

#### (3). Slip flow

In low-permeability formations (less than 0.001 md) or when the pressure is very low, gas slip flow cannot be omitted when studying gas transport in tight reservoirs [6, 24]. Under such kind of flow conditions, gas absolute permeability depends on gas pressure, which can be expressed as follows:

$$\vec{k_{a}}=\vec{k_{i}}\left(1+\frac{b}{p}\right)$$ (14)

where *k*_*a*_ is the apparent permeability, $\vec{k_{i}}$ is the intrinsic permeability, and *b* is the slip coefficient. Some empirical models have been developed to account for slip-flow and Knudsen diffusion in the form of apparent permeability, including correlations developed from the Darcy matrix permeability [8], correlations developed based on flow mechanisms [3, 4, 25], and semi-empirical analytical models by Moridis et al. in 2010 [9]. This research adopted Javadpour’s method, which considered slip flow, viscous flow, and Knudsen diffusion [4]. The apparent permeability is given as follows:

$$\vec{k_{m}}=\vec{k_{mi}}\left(1+\frac{b_{m}}{p_{m}}\right)$$ (15)

$$b_{m}=\frac{16\mu }{3000r}{\left(\frac{8\pi RT}{M}\right)}^{0.5}+{\left(\frac{8\pi RT}{M}\right)}^{0.5}\frac{\mu }{r}(\frac{2}{\alpha }-1)$$ (16)

where *α* is the tangential momentum accommodation coefficient (TMAC), which characterizes the slip effect. *α* is a function of wall surface smoothness, gas type, temperature, and pressure, which varies from 0 to 1 [4, 26, 27]. In the shale matrix with pores in nano to micro scale, it is necessary to consider the effect of slip effect and Knudsen diffusion. Cao et al. (2005) also pointed out that when the gas free mean path is close to the pore radius, the flow falls in the slip regime [28]. The TMAC *α* in the Eq (16) can be used to characterize the slip effect or rarefaction effect. TMAC represents the part of gas molecules reflected diffusely from the tube wall relative to specular reflection [4]. The slip length can be related to the TMAC using equation $l=\frac{2-\alpha }{\alpha }Kn$ [28]. From this we can find that under a certain Kn, the smaller the TMAC *α*, the larger the slip length. More gas molecules will under slip flow regime. Arkilic et al. obtained the *α* about 0.8 for light gases flowing in silicon microchannel [28, 29]. Javadpour has also employed 0.8 as the value for tangential momentum accommodation coefficient in deriving gas permeability in shale matrix [4]. For simplification and consistency, we also employ 0.8 as the TMAC value in this paper. The accurate TMAC value for a specific gas in the mudrock can be obtained using lab experiments or numerical methods, such as Molecular Dynamics method, Monte Carlo method, and direct simulation Monte Carlo method, etc.

So, the flow vector term in the matrix is: ${\left(\nabla \cdot (\rho \;\vec{u})\right)}_{m}=-\nabla .\left(\rho_{gm}(\frac{\vec{k_{m}}}{\mu }\nabla p_{m})\right)=-\nabla .\left(\gamma p_{m}(\frac{\vec{k_{m}}}{\mu }\nabla p_{m})\right)$

For the matrix system, it should be noted that with the gas desorption from the pore wall, the pore radius increases. The volume of gas desorbed from the wall can be characterized by the Langmuir isotherm curve. Considering the gas desorption, the effective pore radius can be expressed as follows [18]:

$$r_{eff}=r_{max}-d_{CH_{4}}\frac{P_{m}/P_{L}}{1+P_{m}/P_{L}}$$ (17)

**Fig 5:**
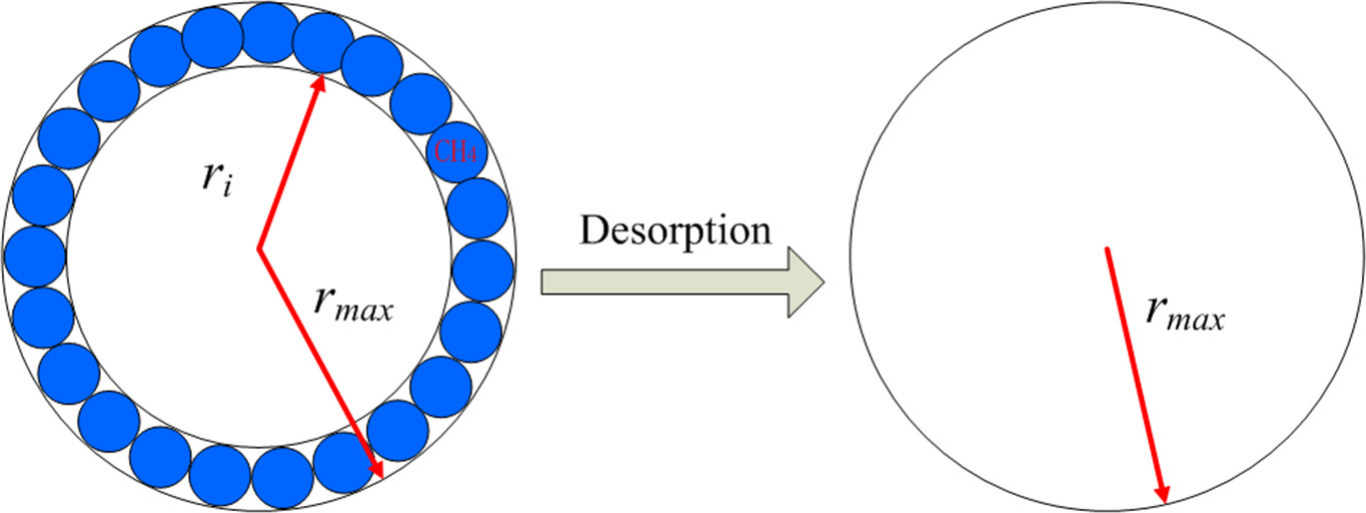
Pore radius change due to gas desorption. If single molecule gas desorption Langmuir isothermal is considered, when all the molecules have desorped from the surface, then the pore radius will increase as shown in the right part.

Here, *r*_*eff*_ is the initial pore radius, $d_{CH_{4}}$ is the molecular diameter of methane, *P*_*L*_ is the Langmuir pressure, and *r*_*max*_ is the maximum pore radius, which is the initial pore radius plus the molecular diameter. With the matrix pressure decreasing during the production process, gas desorbs from the pore wall. So, from Eq (17), we can find that the pore radius is increasing with production. If the matrix pressure can approach 0, which is the ideal scenario, the final pore diameter will be equal to initial radius r_i_. The process is shown in Fig 5.It can be found that after the gas desorbed from the pore wall,the gas transport channel will become larger.Due to the increase of the pore radius,the collision of gas moleculars with the pore wall will become less dominant.Eq (16) has shown this change.With pore radius increase,the slip effect coefficient *b*_*m*_ will decrease. The contribution of Knudsen diffusion and slip flow will contribute less to the gas flux with pore radius increases. At the same time, with the pore radius increase, the viscous flow which is denoted using the blue dots in Fig 4 will become more dominant and contribute more to the gas flux. The total gas flux is the combined effect of these effects.

### Flow vector term in the fracture

In this study, Knudsen diffusion and viscous flow are considered for the gas flow in the frature system. The mass flux can be expressed as the summation of the two mechanisms. Therefore,

$$J_{f}=J_{fv}+J_{fk}=-\frac{\rho_{f}k_{fi}}{\mu_{g}}(\nabla p_{f})-\frac{\rho_{f}D_{kf}}{p_{f}}(\nabla p_{f})$$ (18)

Or

$$J_{f}=-\frac{k_{fi}\rho_{f}}{\mu_{g}}(1+\frac{b_{f}}{p_{f}})(\nabla p_{f})$$ (19)

Using the form of conventional Darcy’s flow equation, the fracture apparent permeability can be expressed as:

$$\vec{k_{f}}=\vec{k_{fi}}\left(1+\frac{b_{f}}{p_{f}}\right)$$ (20)

where

$$b_{f}=\frac{D_{kf}\mu_{g}}{k_{fi}}$$ (21)

$$D_{kf}=\frac{4k_{fi}}{2.8284\sqrt{\frac{k_{fi}}{\varphi_{f}}}}\sqrt{\frac{\pi RT}{2M_{g}}}$$ (22)

where p_f_ is the fracture pressure, k_fi_ is the initial fracture permeability, k_f_ is the apparent fracture permeability, b_f_ is the Klinkenberg coefficient for the fracture system, D_kf_ is the Knudsen diffusion coefficient for the fracture system, and ∅_f_ is the fracture porosity.

Combining all of the above, the flow vector term in the fracture is:

$${\left(\nabla .(\rho \;\vec{u})\right)}_{f}=-\nabla .\left(\rho_{\mathrm{gf}}(\frac{\vec{k_{f}}}{\mu }\nabla p_{f})\right)=-\nabla .\left(\gamma p_{f}(\frac{\vec{k_{f}}}{\mu }\nabla p_{f})\right)$$ (23)

### Source and sink term

For DPCM, it is of great importance to consider the interaction between the matrix and the fracture in simulation. There are different methods for handling this issue in reservoir simulation. The boundary condition approach explicitly calculates the amount of the matrix-fracture petroleum fluid transfer by imposing boundary condition at each time step. It is very useful in well testing [8, 30]. However, this method is impractical in full field simulation due to its high computational cost. Another method is the Warren-Root method [17, 30]. The Warren-Root method calculates the cross flux between the fracture and matrix systems by assuming a pseudo-steady state flow between the matrix and fracture when the no-flow boundary is confined. The gas transfer between the matrix and fracture systems is represented in Eq (24):

$$T=\frac{k_{m}\rho_{g}\sigma (p_{m}-p_{f})}{\mu }$$ (24)

where $\sigma =4\left(\frac{1}{L_{x}^{2}}+\frac{1}{L_{y}^{2}}+\frac{1}{L_{z}^{2}}\right)$ is the crossflow coefficient between the matrix and fracture systems, and L_x_, L_y_, and L_z_ are the fracture spacings in the *x*,*y*, *z* directions, respectively. In the fracture system, there exists a source term that flows into fracture from the matrix and a sink term that flows out of the fracture into the wellbore. Therefore, for the matrix system, the sink term that flows out of the matrix to the fracture can be described as follows:

$$Q_{m}=-T$$ (25)

The sink term followed the model developed by Aronofsky and Jenkins [31], which considers gas production from vertical well and can be expressed using Eq (26):

qp=kfρfμgθ[ln⁡(rerw)+s+Dq](pf−−pwf) (26)

In Eq (26), when the production well is in the corner, θ = π/2. When the production well is in the center, θ = 2π [32]. In addition, p_wf_ is the bottomhole flowing pressure; $\overset{-}{p_{f}}$ is the average pressure in the fracture system; r_w_ is the well radius; and r_e_ is the drainage radius, which can be expressed as follows:

$${\text{r}}_{\text{e}}=\left\{ \begin{array}{l} \;0.14\sqrt{2[{\left(\Delta \text{x}\right)}^{2}+{\left(\Delta \text{y}\right)}^{2}]}\;{\text{k}}_{\text{x}}={\text{k}}_{\text{y}}\\ 0.28\frac{{\left[{\left({\text{k}}_{\text{y}}/{\text{k}}_{\text{x}}\right)}^{1/2}\Delta {\text{x}}^{2}+{\left({\text{k}}_{\text{x}}/{\text{k}}_{\text{y}}\right)}^{1/2}\Delta {\text{y}}^{2}\right]}^{0.5}}{{\left({\text{k}}_{\text{y}}/{\text{k}}_{\text{x}}\right)}^{1/4}+{\left({\text{k}}_{\text{x}}/{\text{k}}_{\text{y}}\right)}^{1/4}}\;{\text{k}}_{\text{x}}\neq {\text{k}}_{\text{y}} \end{array}\right.$$ (27)

where Δx, Δy, k_x_, and k_y_ are the length of grid and permeability in the x and y directions. For the fracture sytem, Q_f_ = T − q_p_.

### Gas viscosity change

Currently, some reservoir simulators have considered gas viscosity change, which is a function of pressure. However, this study considered the gas viscosity change to be a function of the Knudsen number (the ratio of the free molecular length to the pore diameter), which is dependent on the gas transport period, as discussed previously. Gas viscosity changes with the Knudsen number in the production process [33].

**Fig 6:**
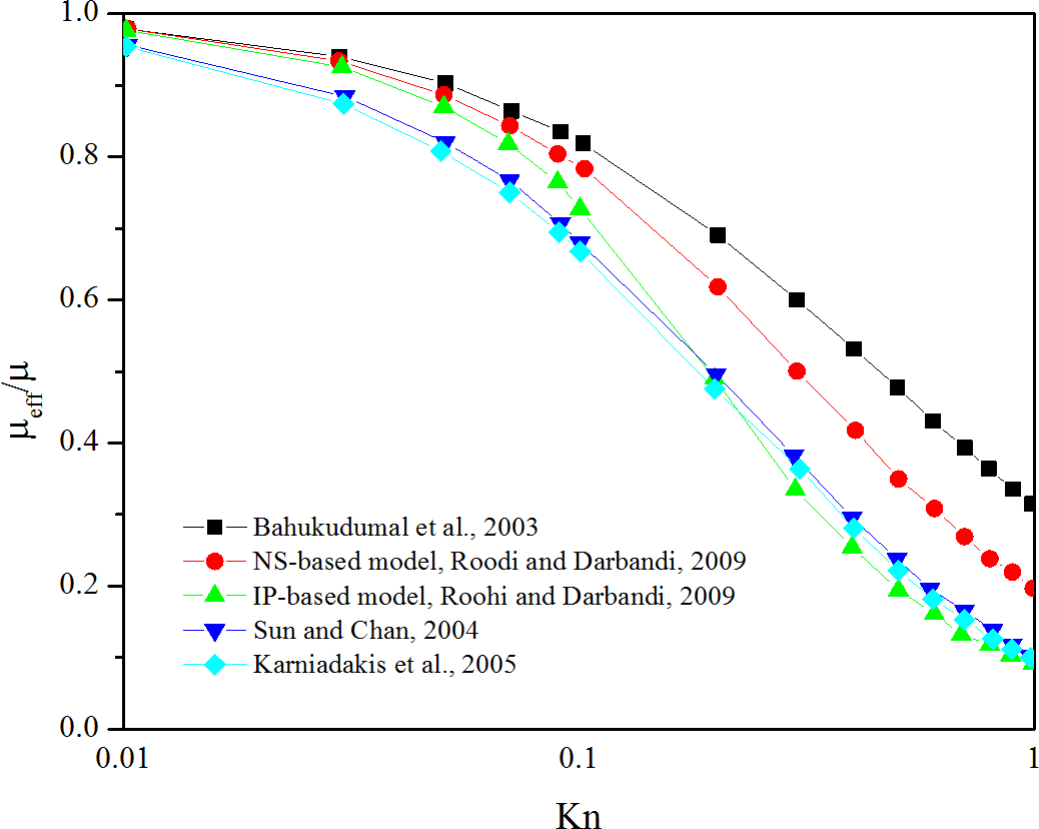
Gas viscosity variation with the Knudsen number (from 0.01 to 1), also means from slip flow to transition flow. Gas viscosity has changed a lot when the Knudsen number changes, which means it is necessary to consider the gas viscosity variation (Modified from [33]).

Fig 6 shows that the ratio of the effective viscosity to the initial viscosity changes with the Knudsen number, which is a function of gas pressure. Eq (28) shows the relationship between gas viscosity and the Knudsen number. Beskok and Karniadakis (1999) have derived the expression of the Knudsen number using a more general form, as shown in Eq (29), which is easier to be considered in the numerical simulation [16]. Also, the results of this analytical equation has good agreement with DSMC results and with the linearized Boltzmann solution.

$$\mu_{\text{gm}}=\mu_{0}\frac{1}{1+{\mathrm{\beta K}}_{\text{n}}};\;\mu_{\text{gf}}=\mu_{0}\frac{1}{1+{\mathrm{\beta K}}_{\text{n}}}$$ (28)

where μ_0_ is the initial viscosity at the initial reservoir pressure (*p*_*m*_ *= p*_*f*_ *= p*_*i*_). This suggested a Knudsen dependence of the rarefaction parameter *β* and provided an analytical expression for this dependence. Eq (29) expresses the definition of the Knudsen number, which can be related to be the basic parameters that change with pressure:

$${\text{K}}_{\text{n}}=\frac{\mu_{\text{g}}}{4\text{p}}\sqrt{\frac{\mathrm{\pi RT}\phi_{\text{m}}}{{\text{M}}_{\text{g}}{\text{K}}_{\text{i}}}}$$ (29)

The final mathematical model to characterize gas transport in SGRs can be expressed as follows:

$$\mathrm{Matrix}:\;\left[\gamma \phi_{m}+\frac{(1-\phi_{m})M_{g}p_{L}V_{L}\rho_{s}}{V_{\mathrm{std}}{\left(p_{L}+p_{m}\right)}^{2}}\right]\frac{\partial p_{m}}{\partial t}-\nabla \cdot \left[\gamma [\frac{k_{\mathrm{mi}}(p_{m}+b_{m})}{\mu_{g}}(\nabla p_{m})]\right]=-T$$ (30)

$$\mathrm{Fracture}:\;[\gamma \phi_{f}]\frac{\partial p_{f}}{\partial t}-\nabla \cdot \left[\gamma [\frac{p_{f}k_{f}(p_{f}+b_{f})}{\mu_{g}}(\nabla p_{f})]\right]=T-q_{p}$$ (31)

With following boundary conditions considered in this paper:

$$\mathrm{Initial condition}:\;{p_{m}|}_{t=0}={p_{f}|}_{t=0}=p_{i}$$ (32)

$$\mathrm{Boundary condition for matrix}:\;{F_{m}\cdot n|}_{\Gamma_{1}}=0\;(\frac{\partial p}{\partial n}{|}_{\Gamma_{1}}=0)$$ (33)

$$\mathrm{Boundary condition for fracture}:\;{F_{f}\cdot n|}_{\Gamma_{1}}=0\;(\frac{\partial p}{\partial n}{|}_{\Gamma_{1}}=0),\;{p_{f}(x,y,t)|}_{\Gamma_{2}}=p_{w}$$ (34)

In this paper, the model Eqs (30)–(34) and four versions of its simplification have been considered: (1) considering the basic DPM, which has considered none of adsorption, non-Darcy flow, gas viscosity, and pore radius change. (2) considering the basic DPM with adsorption which corresponds to the first term of the left part of Eq (30); (3) considering the basic DPM with adsorption and non-Darcy permeability change. That is, *b*_*m*_ ≠ 0 and *b*_*f*_ ≠ 0; (4) considering the basic DPM with adsorption, non-Darcy permeability change, and gas viscosity change which is represented in the Eq (28); and (5) considering adsorption, non-Darcy permeability change, gas viscosity change, and pore radius change. Pore radius change is represented in Eq (17), in which *r*_*eff*_ represents the changing radius as shown in *b*_*m*_ (*r* = *r*_*eff*_ in Eq (16)).

The finite element method was used to solve the PDE equations from Eqs (30)–(34), which are listed above. First, multiply a test function *v*(*x*,*y*) on both sides of original Eq (30) where *Ω* is the domain; *Γ*_1_ is outside boundary; and *Γ*_2_ is the inner boundary.

$$[\gamma \phi_{m}+\frac{(1-\phi_{m})M_{g}p_{L}V_{L}\rho_{s}}{V_{\mathrm{std}}{\left(p_{L}+p_{m}\right)}^{2}}]\frac{\partial p_{m}}{\partial t}v-\nabla \cdot [\gamma [\frac{k_{\mathrm{mi}}(p_{m}+b_{m})}{\mu_{g}}(\nabla p_{m})]]v=-\mathrm{Tv}$$ (35)

$$\int_{\Omega }[\gamma \phi_{m}+\frac{(1-\phi_{m})M_{g}p_{L}V_{L}\rho_{s}}{V_{\mathrm{std}}{\left(p_{L}+p_{m}\right)}^{2}}]\frac{\partial p_{m}}{\partial t}v\;\mathrm{dxdy}-\int_{\Omega }\nabla \cdot [\gamma [\frac{k_{\mathrm{mi}}(p_{m}+b_{m})}{\mu_{g}}(\nabla p_{m})]]v\;\mathrm{dxdy}=\int_{\Omega }-\mathrm{Tv}\;\mathrm{dxdy}$$ (36)

Using Green’s Theorem (Divergence Theory and Integration by parts in multi-dimension), we obtain that:

$$\int_{\Omega }\nabla \cdot [\gamma [\frac{k_{\mathrm{mi}}(p_{m}+b_{m})}{\mu_{g}}(\nabla p_{m})]]v\;\mathrm{dxdy}=\int_{\partial \Omega }[\gamma [\frac{k_{\mathrm{mi}}(p_{m}+b_{m})}{\mu_{g}}(\nabla p_{m})]\cdot \vec{n}]v\;\mathrm{ds}-\int_{\Omega }\gamma [\frac{k_{\mathrm{mi}}(p_{m}+b_{m})}{\mu_{g}}]\nabla p_{m}\cdot \nabla \mathrm{vdxdy}$$ (37)

$$\int_{\Omega }[\gamma \phi_{\text{m}}+\frac{(1-\phi_{\text{m}}){\text{M}}_{\text{g}}{\text{p}}_{\text{L}}{\text{V}}_{\text{L}}\rho_{\text{s}}}{{\text{V}}_{\text{std}}{\left({\text{p}}_{\text{L}}+{\text{p}}_{\text{m}}\right)}^{2}}]\frac{\partial {\text{p}}_{\text{m}}}{\partial \text{t}}\text{v}\;\text{dxdy}-\int_{\partial \Omega }\left[\gamma \left[\frac{{\text{k}}_{\text{mi}}({\text{p}}_{\text{m}}+{\text{b}}_{\text{m}})}{\mu_{\text{g}}}(\nabla {\text{p}}_{\text{m}})\right]\cdot \vec{\text{n}}\right]\text{v ds}+\int_{\Omega }\gamma \left[\frac{{\text{k}}_{\text{mi}}({\text{p}}_{\text{m}}+{\text{b}}_{\text{m}})}{\mu_{\text{g}}}\right]\nabla {\text{p}}_{\text{m}}\cdot \nabla \text{vdxdy}=\int_{\Omega }-\text{Tv dxdy}$$ (38)

If we just consider the solution on the domain *Ω*, the weak form for the governing Eq (30) is:

$$\int_{\Omega }[\gamma \phi_{\text{m}}+\frac{(1-\phi_{\text{m}}){\text{M}}_{\text{g}}{\text{p}}_{\text{L}}{\text{V}}_{\text{L}}\rho_{\text{s}}}{{\text{V}}_{\text{std}}{\left({\text{p}}_{\text{L}}+{\text{p}}_{\text{m}}\right)}^{2}}]\frac{\partial {\text{p}}_{\text{m}}}{\partial \text{t}}\text{v}\;\text{dxdy}+\int_{\Omega }\gamma \left[\frac{{\text{k}}_{\text{mi}}({\text{p}}_{\text{m}}+{\text{b}}_{\text{m}})}{\mu_{\text{g}}}\right]\nabla {\text{p}}_{\text{m}}\cdot \nabla \text{vdxdy}=\int_{\Omega }-\text{Tv dxdy}$$ (39)

Taking the similar procedures on governing Eq (31), we obtain:

$$\int_{\Omega }[\gamma \phi_{\text{f}}]\frac{\partial {\text{p}}_{\text{f}}}{\partial \text{t}}\text{v}\;\text{dxdy}+\int_{\Omega }\gamma \left[\frac{{\text{p}}_{\text{f}}{\text{k}}_{\text{f}}({\text{p}}_{\text{f}}+{\text{b}}_{\text{f}})}{\mu_{\text{g}}}\right]\nabla {\text{p}}_{\text{m}}\cdot \nabla \text{vdxdy}=\int_{\Omega }\left(\text{T}-{\text{q}}_{\text{p}}\right)\text{v dxdy}$$ (40)

## Model Verification

**Fig 7:**
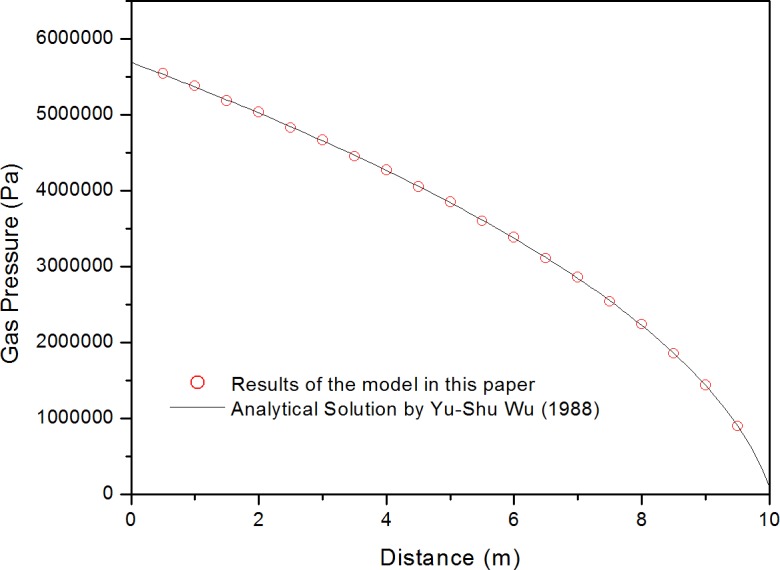
Comparison between analytical solution and numerical simulation in this paper.

As there is no real field data which is same with this therotical case, a common method to verify the righteousness is to compare the results of the numerical simulation against the analytical results. Wu et al. derived the analytical solution for 1D steady-state gas transport [34], which is shown in Eq (41). Thus, we can verify our model by comparing the results from simpilification version of the model in this paper with the analytical results. The reservoir properties and Klinkenberg properties used in this verification were obtained from the experimental study of the welded tuff at Yucca Mountain.

$$p(x)=-b+\sqrt{b^{2}+(p{\left(L\right)}^{2}+2b[(p(L)])+\frac{2q_{m}\mu_{g}(L-x)}{k_{\infty }\beta }}$$ (41)

where *b* is the Klinkenberg factor, 7.6×10^5^ Pa; *p*(*L*) is the gas pressure at the outlet (x = L), 1.0 × 10^5^ Pa; *q*_*m*_ is the air injection rate, 1.0 × 10^−6^ kg/s; *μ*_*g*_ is the gas dynamic viscosity, 1.84 × 10^−5^ Pa.s; *L* is the length from the inlet (x = 0) to the outlet, 10 m; x is the task location along the gas transport path, m; k_∞_ is the intrisic permeability for welded tuff atYucca Mountain, 5.0 × 10^−19^ m^2^; and *β* is the compressibility factor, 1.8 × 10^−5^ Pa^-1^m^-1^. Fig 7 presents the match results between the model presented in this paper and the analytical solution. As shown in Fig 7, the results obtained using the model presented in this paper agrees well with the analytical solution [34] provided by Wu et al., which validated our mathematical model.

## Results and Analysis

**Fig 8:**
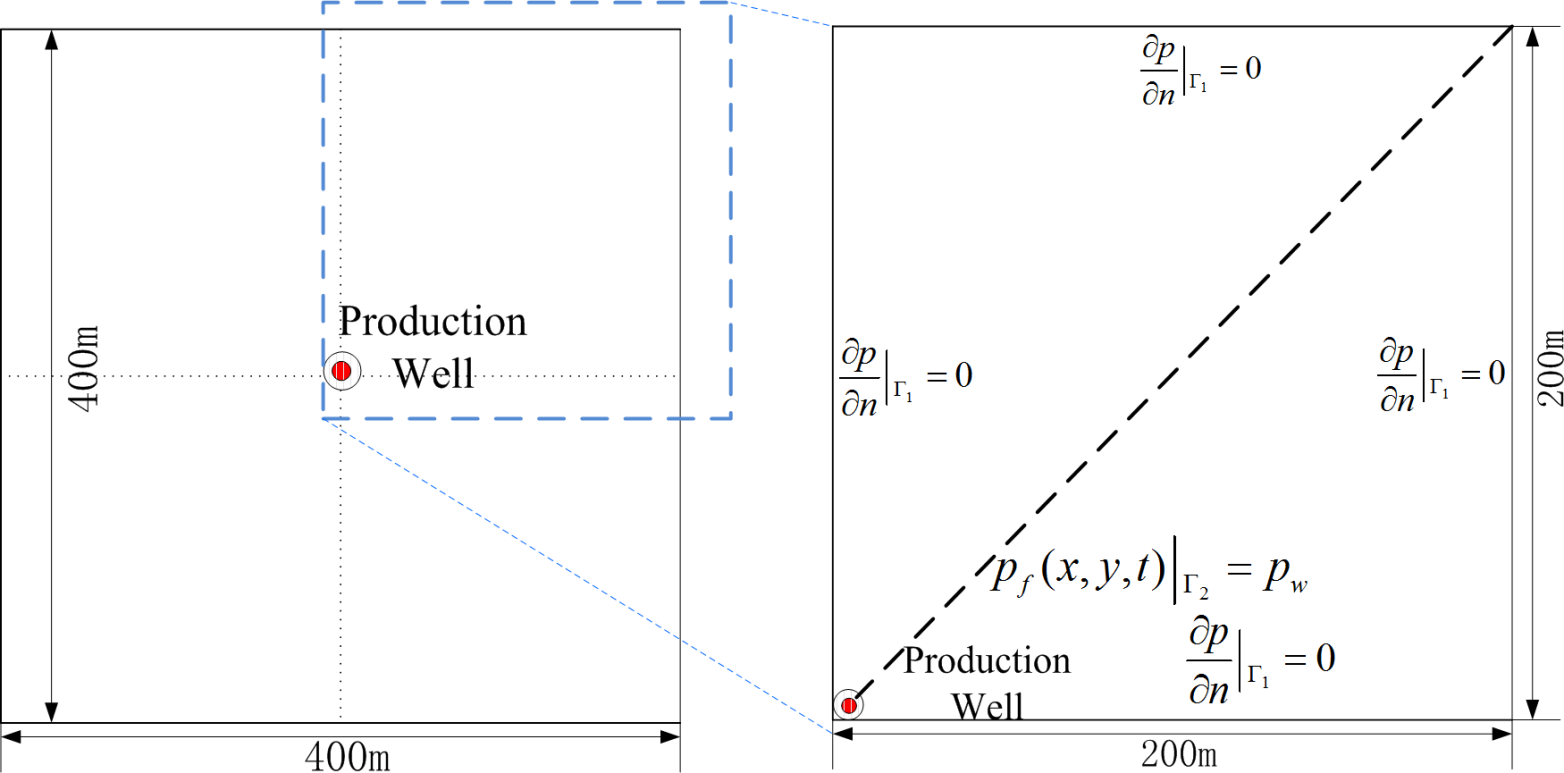
Real reservoir and simplified 2-D reservoir simulation model.

The 2-D reservoir model is shown in Fig 8. For simplification, we only studyed the ¼ area of the whole reservoir. The reservoir parameters are shown in Table 1. The parameters used in this paper were selected based on the literature review regarding shale gas simulation [10]. The simulated reservoir is located at a depth of 5463 ft with a pressure gradient of 0.53 *psi*/*ft* and a temperature gradient of 0.065 *K* / *ft*, which corresponded to the initial reservoir pressure and reservoir temperature of 20 *MPa* and 353.15 *K*, respectively. Other parameters are listed in Table 1.

**Table 1:** Parameters used in the simulation model.

Parameter	Value	Unit	Definition
*D*	5463	*ft*	reservoir depth
*G*_*p*_	0.54	*psi*/*ft*	reservoir pressure gradient
*G*_*T*_	0.059	*K* / *ft*	reservoir temperature gradient
k_*mi*_	0.04	*md*	matrix initial permeability
k_*fi*_	10	*md*	fracture initial permeability
*ϕ*_*m*_	0.05	Dimensionless	matrix porosity
*ϕ*_*f*_	0.001	Dimensionless	fracture porosity
R	8.314	*P*_*a*_ * *m* ^3^ / (*mol* * *K*)	gas constant
z	1	Dimensionless	gas compressibility factor
*p*_*i*_	10.4	*MP*_*a*_	initial reservoir pressure
*p*_*w*_	3.45	*MP*_*a*_	bottom hole pressure
*M*_*g*_	0.016	*kg*/*mol*	mole weight of CH_4_
*V*_*std*_	0.0224	*m* ^3^/*mol*	standard gas volume
*p*_*L*_	2.07	*MP*_*a*_	langmuir pressure
*V*_*L*_	2.83 × 10^−3^	*m* ^3^/*kg*	langmuir volume
*ρ*_*s*_	2550	*kg*/*m* ^3^	shale rock density
*μ*_*g*_	1.02 × 10^−5^	*P*_*a*_ * *s*	initial gas viscoisty
*r*_*w*_	0.1	*m*	wellbore radius
*L*_*x*_	0.2	*m*	fracture spacing

In this study, firstly we investigated the impact of parameters such as the initial reservoir pressure, matrix permeability, fracture permeability, matrix porosity, and fracture porosity on shale gas production. Then, the mechanisms discussed earlier were gradually incorporated into the simulation model to investigate their impact on gas transport in the shale strata. These mechanisms are adsorption, permeability change due to comprehensive slip effect, viscosity change, and radius change due to gas adsorption.

### Effect of Gas adsorption

**Fig 9:**
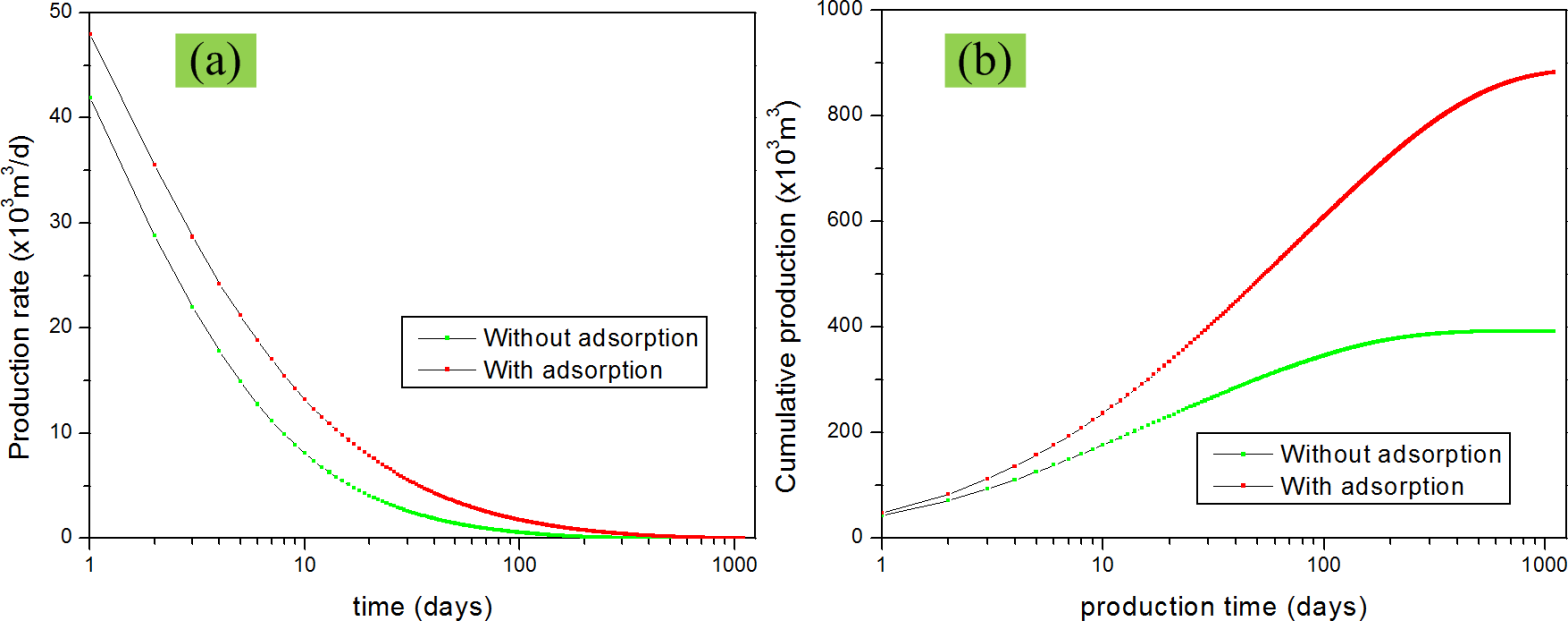
Effect of adsorption on gas production performance. (a) production rate vs. time; (b) cumulative production vs. time.

In this study, we compared scenarios in which adsorption was considered and was not considered. When adsorption was considered, there is an adsorption term on the left hand side of Eq (30). Only free gas is considered in the matrix system if adsorption is ignored. The following analysis are all based on the scenario in which adsorption was considered. First, we need to know the effect of adsorption on the gas production. Fig 9 illustrates the effect of adsorption on the production rate and cumulative production. It is obvious that adsorption has a great effect on the production rate and cumulative production. Ignoring the effect of adsorption gas leads to great underestimations in the gas production in such situations.

### Effect of non-Darcy flow

**Fig 10:**
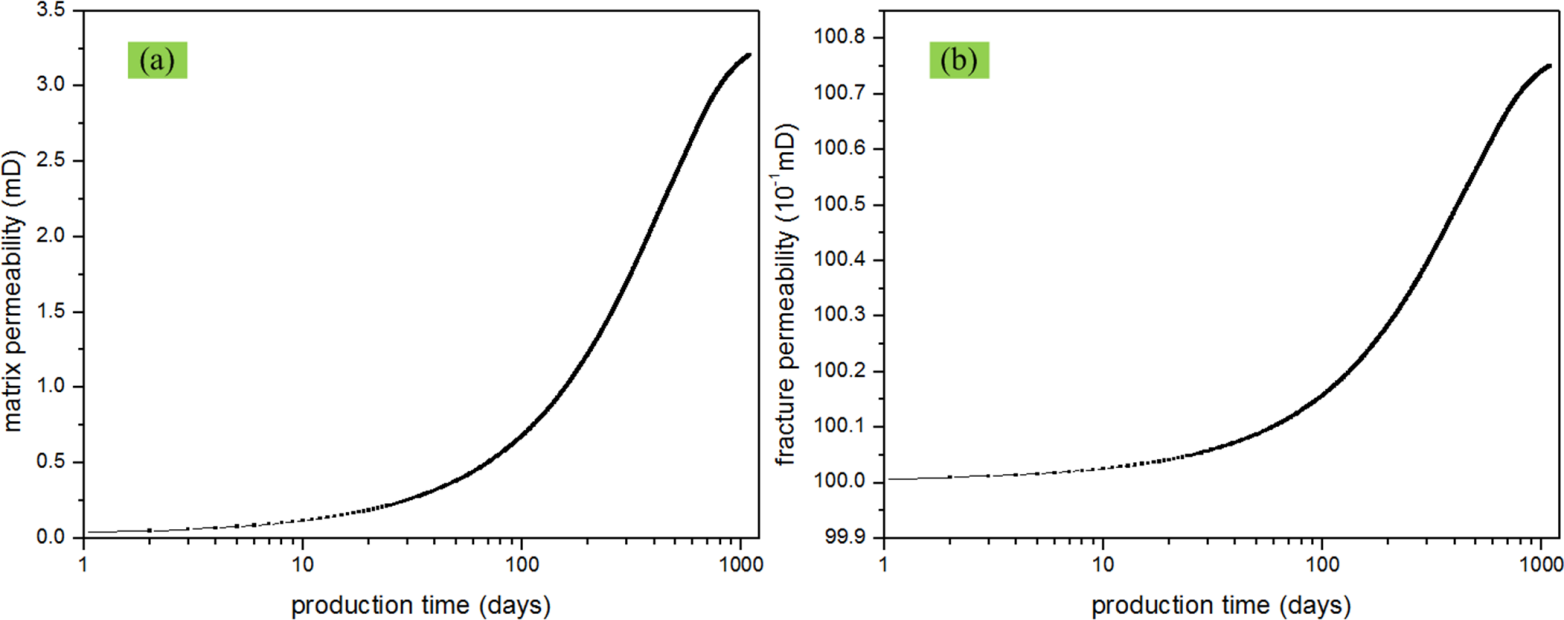
Matrix and fracture permeability change with time. (a) matrix permeability vs. time; (b) fracture permeability vs. time.

**Fig 11:**
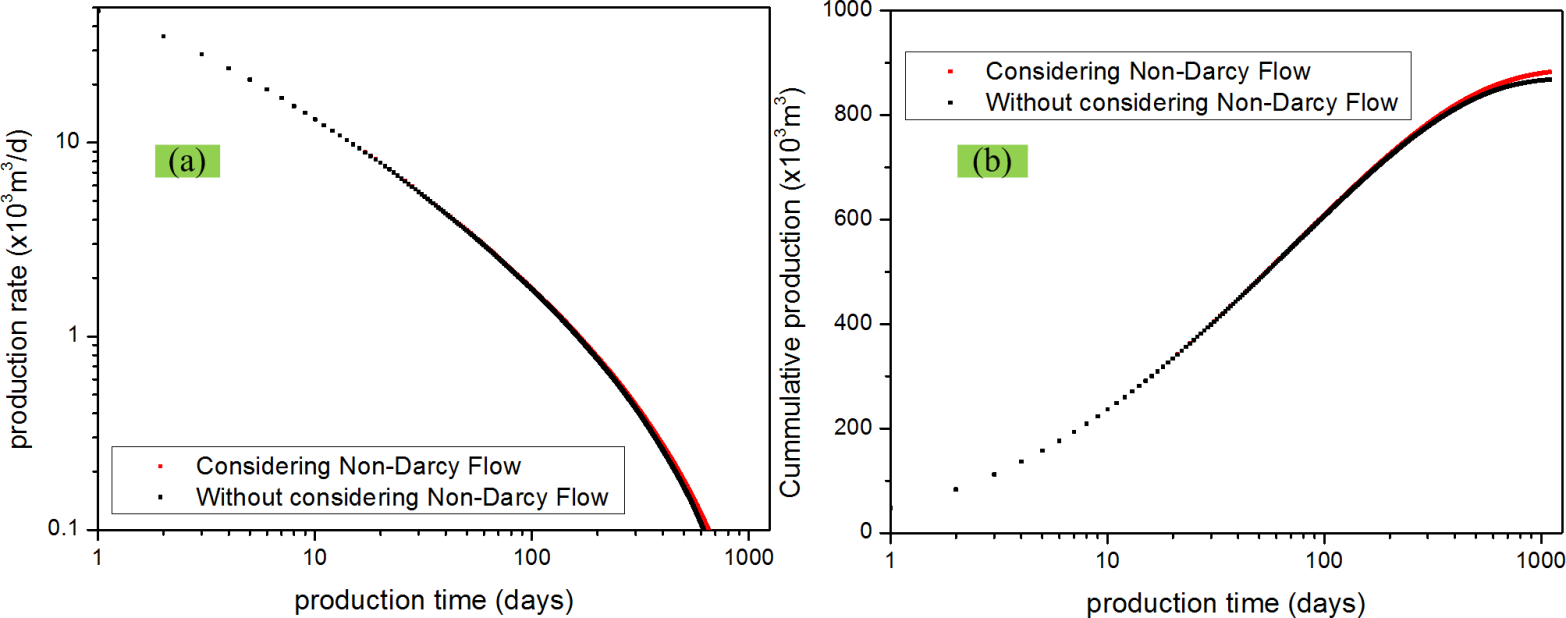
Effect of Non-Darcy flow on gas production performance. (a) production rate vs. time; (b) cumulative production vs. time.

Effect of non-Darcy flow can be considered by adjusting the slip coefficient which appereas in Eq (30). If non-Darcy flow is considered, the slip coefficient *b*_*m*_ = *b*_*f*_ = 0. Fig 10 illustrates the matrix permeability and fracture permeability change with time. From the Fig 10, it is clear that the gas permeabilities in the matrix and fracture gradually increase during pressure depetion. The matrix permeability has a greater increase then the fracture permeability. This result has also validated the righteous of our model. The fluid flow channels are larger in the fractures than those in matrix. So, the slip effect coefficient in the fracture (*b*_*f*_) will be smaller compared with those in matrix (*b*_*m*_). So, the change of gas permeability will also be small. As shown in Fig 11, the impact of non-Darcy on the production rate and the cumulative production are plotted on the log-log scales. It is clear that considering non-Darcy flow increases the production rate and cumulative production; however, there is not a significant difference between these two scenarios. This is because though matrix permeability increases a lot as shown in Fig 10(A), however, fracture permeability is critical in determining fliud flow rate from the fracture system to the wellbore. Therefore, it is important to increase the fracture permeability rather than matrix permeability by including hydraulic fractures in using stimulations.

### Effect of gas viscosity change

**Fig 12:**
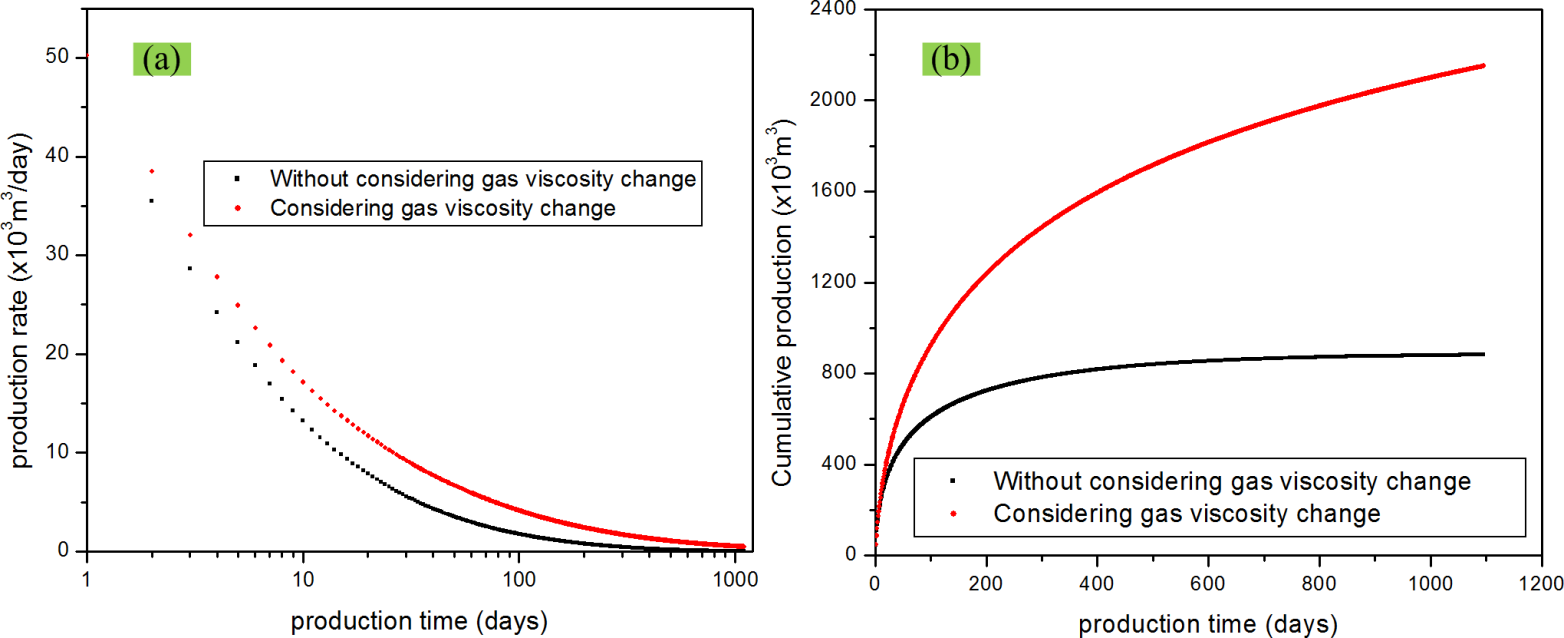
Effect of gas viscosity change on gas production. (a) production rate vs. time; (b) cumulative production vs. time.

Effect of gas viscosity change was evalutaed on the basis of the previous study, which considered adsorption and non-Darcy flow. That is, if the gas viscosity change is considered, then *b*_*m*_ ≠ 0, *b*_*f*_ ≠ 0. The gas viscosity change is represented in Eq (28). If the gas viscosity change is not considered, then *b*_*m*_ ≠ 0, *b*_*f*_ ≠ 0, and the gas viscosity is a constant. As shown before, the gas viscosity decreases with production and pressure depletion. Fig 12 shows the comparison of the production rate and cumulative production between these two scenarios. It can be seen that gas viscosity has a great impact on the production rate and cumulative production under the production condition of this paper. From Eq (28), we can find that gas viscosity is a non-linear function of the pressure. During the gas production process, pressure decrease leads to a decrease in the gas viscosity, which results in an increase in the production. Therefore, ignoring the effect of gas viscosity change decreases the estimated production.

### Effect of pore radius change

**Fig 13:**
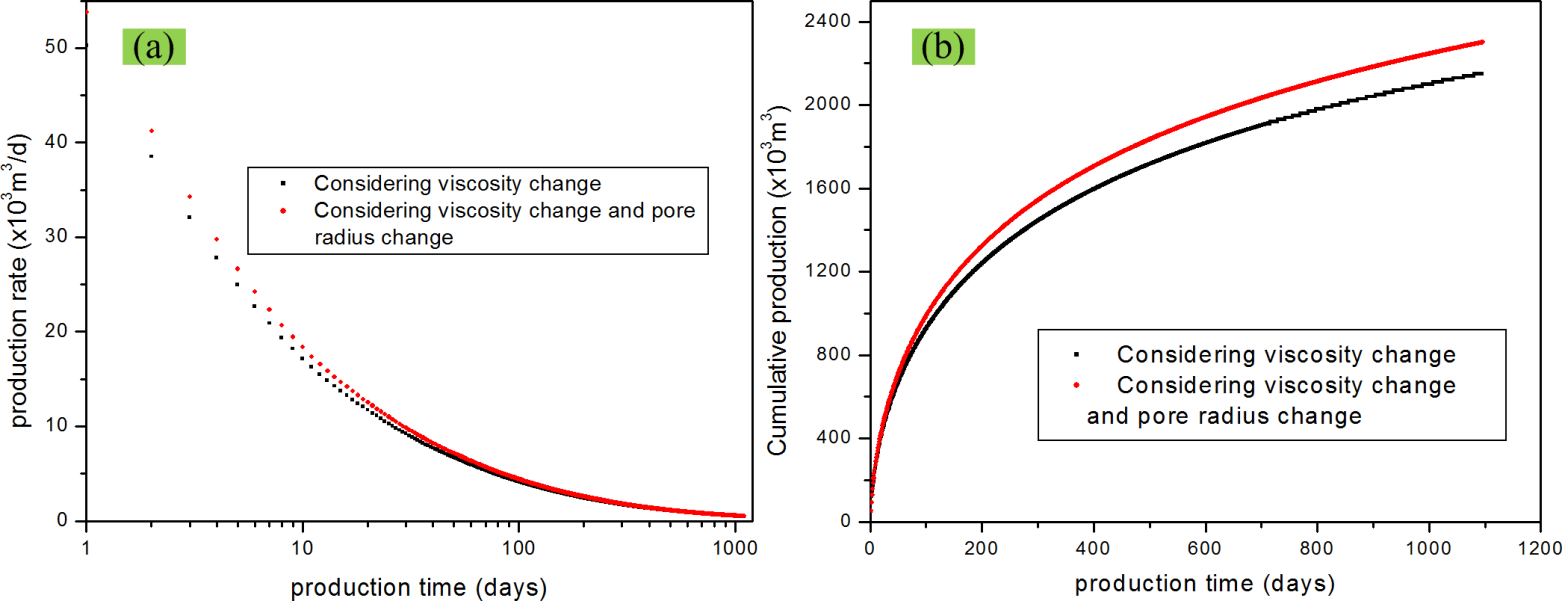
Effect of pore radius increase due to gas desorption on gas production. (a) production rate vs. time; (b) cumulative production vs. time.

Pore radius change is represented in Eq (17), which will change the radius shown in the part of *b*_*m*_ (*r = r*_*eff*_) in Eq (16). All the factors that have been considered before are still considered when analyzing the effect of pore radius change: adsorption, non-Darcy flow, and gas viscosity change. From Eq (17), we can find that the maximum pore radius in the production will be intrinsic radius plus the diameter of CH_4_. Therefore, the pore radius actually does not change too much. However, *r*_*eff*_ is affected by the matrix pressure, which changes during reservoir depletion; therefore, it is still important to consider this effect. The effect of the pore radius change on the production rate and cumulative production are shown in Fig 13. From Fig 13, we can find that although there is a slight increase in the production rate and cumulative production. The effect of the radius change is not very significant.

**Fig 14:**
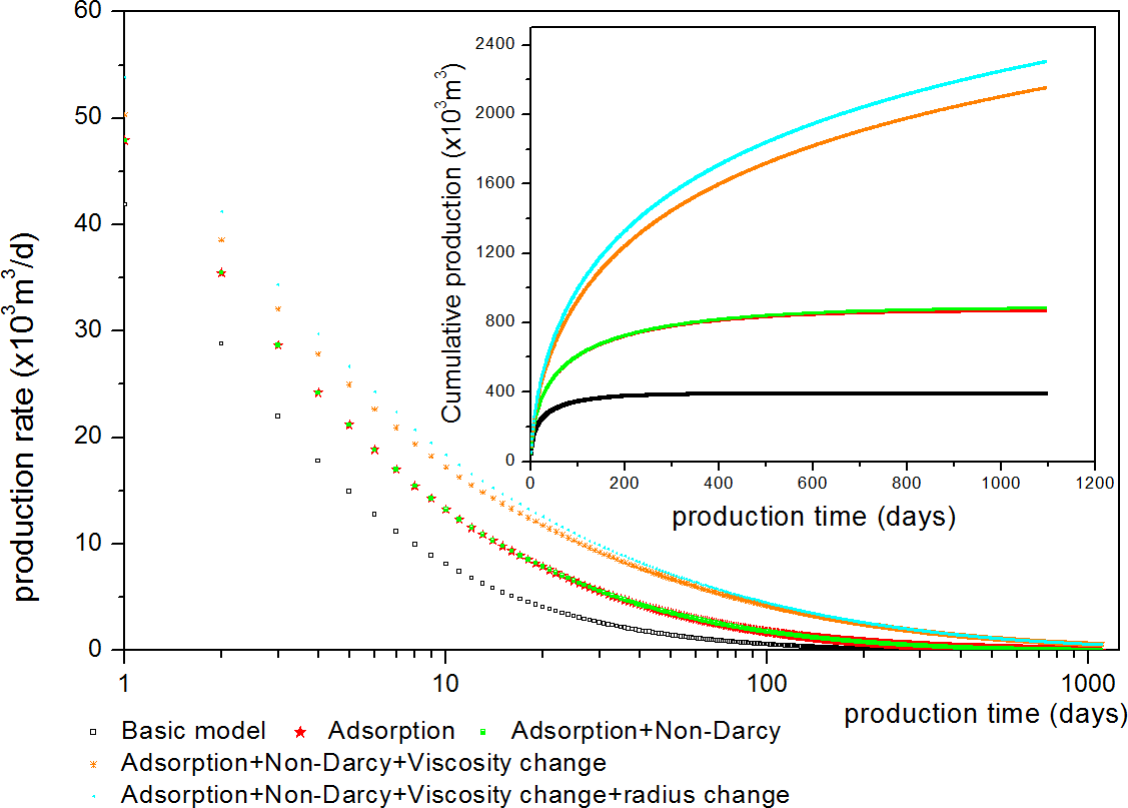
Comparison of five different models: (1) basic model; (2) Considering adsorption; (3) Considering adsorption and non-Darcy permeability change; (4) Considering adsorption and non-Darcy permeability change, gas viscosity change; (5) Considering adsorption, non-Darcy permeability change, gas viscosity change and pore radius change.

Fig 14 shows the comparison of the above 5 models: (1) basic DPM which has not considered adsorption, non-Darcy flow, gas viscosity, and pore radius change; (2) Considering adsorption; (3) Considering adsorption and non-Darcy permeability change; (4) Considering adsorption, non-Darcy permeability change, and gas viscosity change; (5) Considering adsorption, non-Darcy permeability change, gas viscosity change, and pore radius change. It can be found that the mechanisms that have considered will increase the production. Ignoring any one of these factors will lead to an underestimated gas production.

## Sensitivity Analysis

To study the effect of reservoir parameters on gas production, we used the model that considered the effect of adsorption and non-Darcy flow. The effect of the following reservoir parameters was analyzed. (1) initial pressure; (2) matrix permeability; (3) fracture permeability; (4) matrix porosity; and (5) fracture porosity. For each scenario, we compared the production rate and cumulative production for three stages. In this study, when discussing the effect of certain parameters, the other parameters were kept the same as those listed in the Table 1.

### Effect of initial pressure

**Fig 15:**
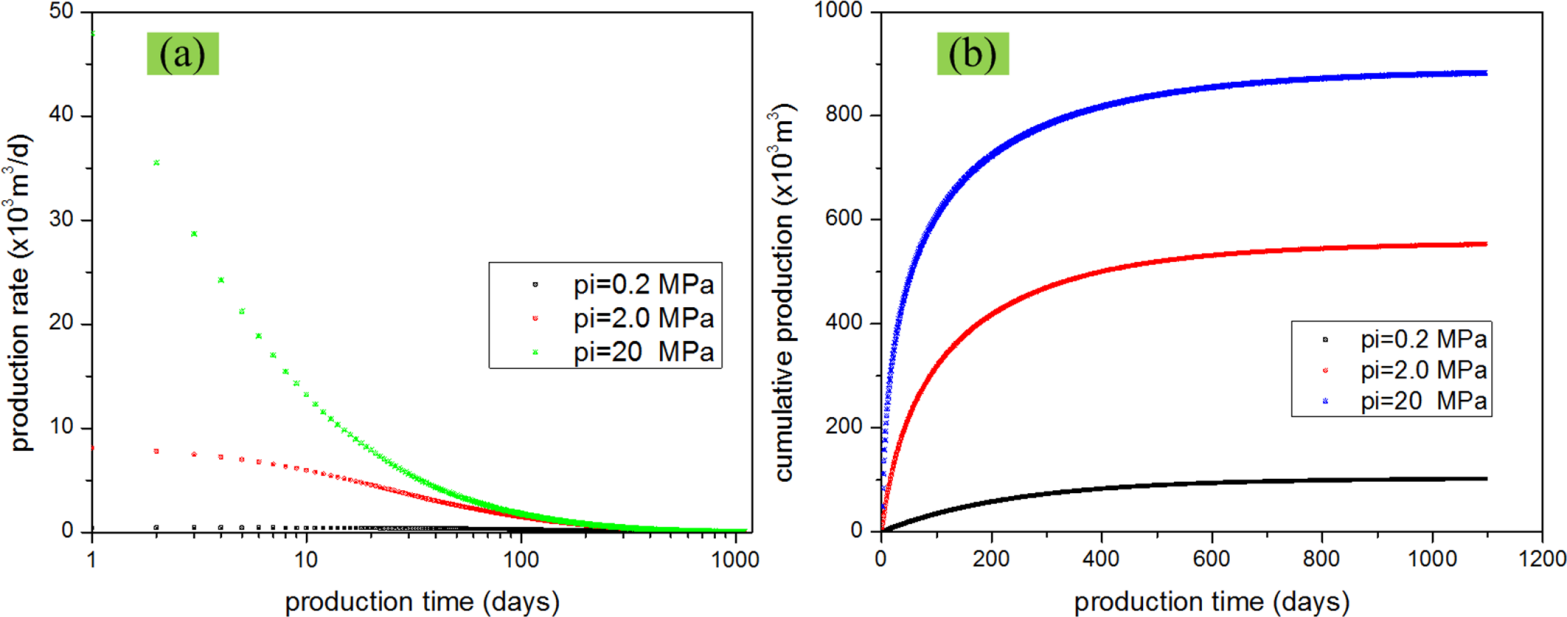
Effect of initial reservoir pressure on gas production. (a) production rate vs. time; (b) cumulative production vs. time.

Initial reservoir pressure is very important in the production, especially for gas reservoir which has no other driving force. Here, we analyzed the effect of the initial pressure on gas production. We considered the scenarios of the initial pressure being equal to 0.2 MPa, 2.0 MPa, and 20 MPa. Other parameters were kept the same as those listed in the Table 1. Fig 15 shows the comparison of the production rate and cumulative production under different initial reservoir pressures. From the plots, we can find that initial pressure has a great effect on the gas production; there is 3- to 5- fold increase in the cumulative production when the intial pressure increases 10-fold. With the increase of reservoir initial pressure, gas production rate and gas cumulative production increase.

### Effect of matrix and fracture permeability

**Fig 16:**
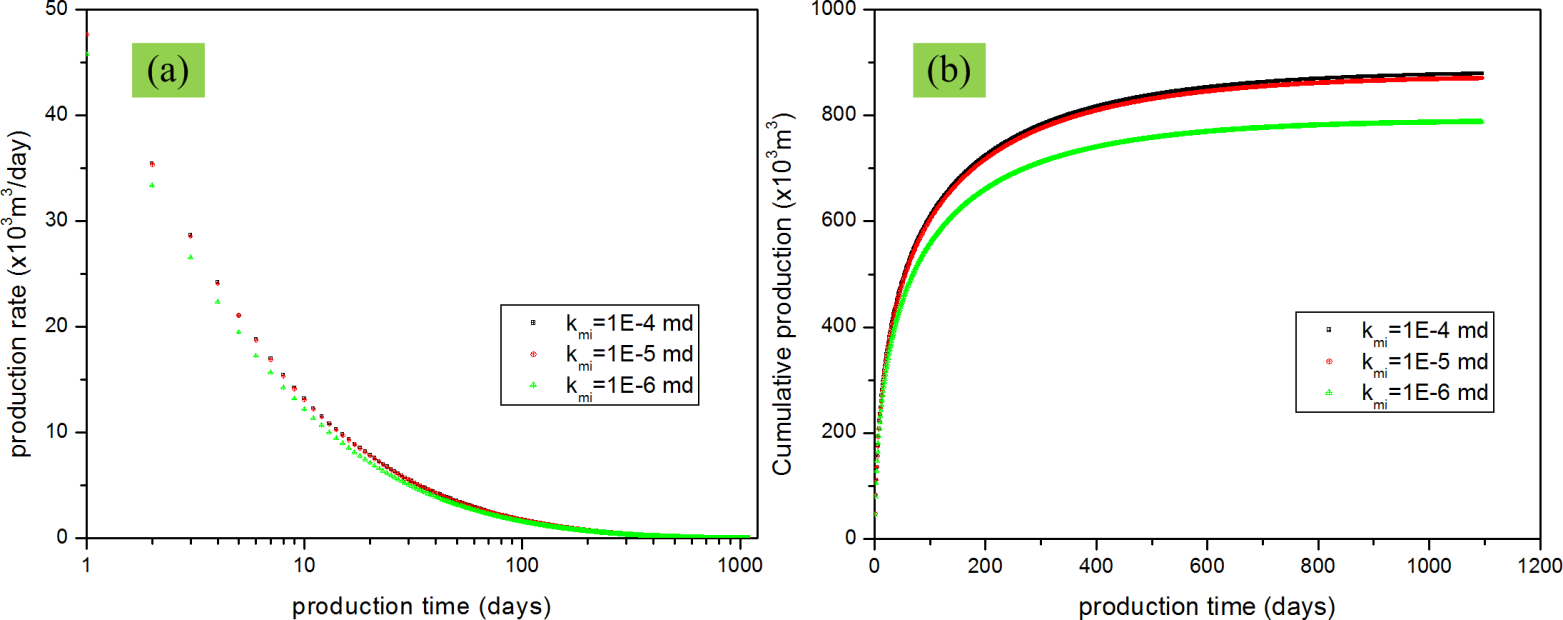
Effect of matrix permeability on gas production. (a) production rate vs. production time; (b) cumulative production vs. production time.

**Fig 17:**
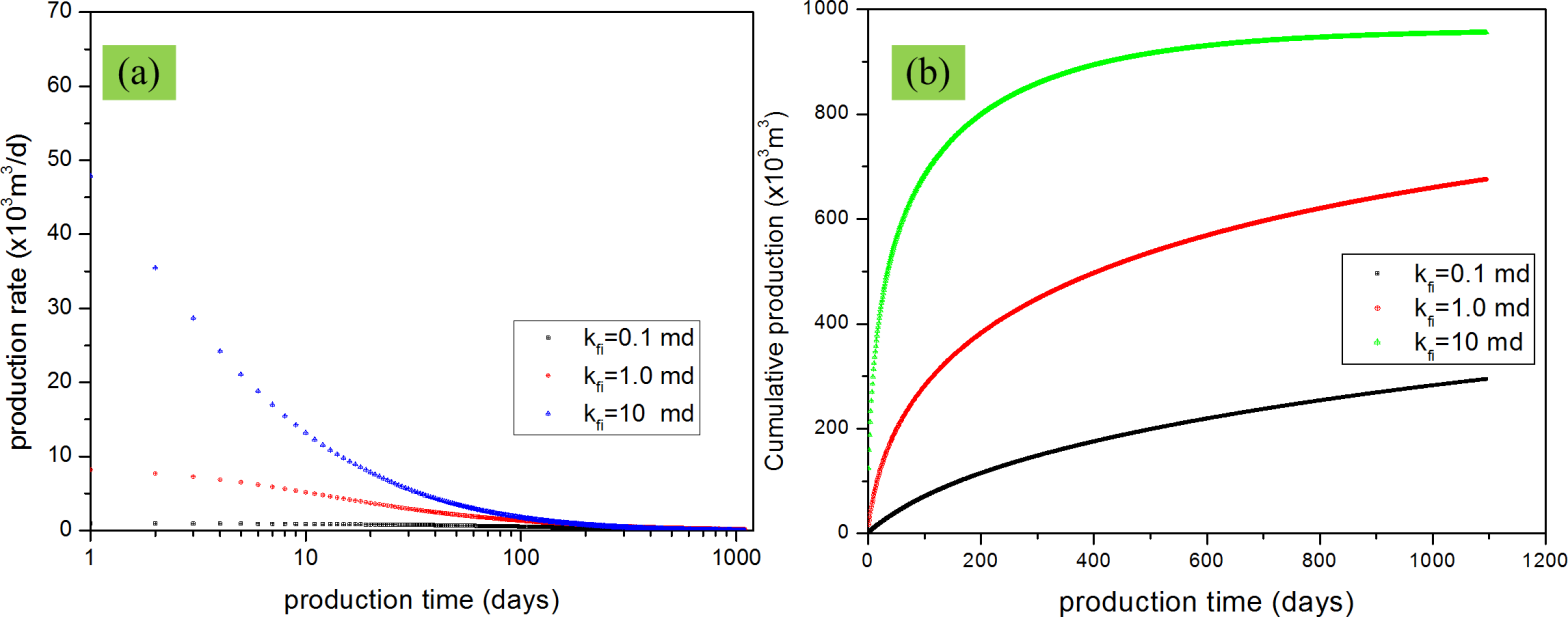
Effect of fracture permeability on gas production. (a) production rate vs. production time; (b) cumulative production vs. production time.

Permeability is the king in the reservoir production. For DPM, matrix permeability and fracture permeability are not the same and the effects of these two kinds of permeability are also different. In this study, the effects of matrix permeability and fracture permeability on gas production were evaluated together to show which effect will be more important. Fig 16 shows the effects of matrix permeability when the permeability is varied from 1.0×10^−4^ mD to 1.0×10^−5^ mD to 1.0×10^−6^ mD. Fig 17 shows the effects of fracture permeability when the permeability changes from 0.1 mD to1 mD to 10 mD. From these figures, we can find that the production rate and cumulative production increase when the matrix permeability or fracture permeability increases. However, from the comparison between Fig 16(A) and Fig 16(B), and between Fig 17(A) and Fig 17(B), we can find that increasing fracture permeability has a more apparent effect on the production rate and cumulative production compared with the matrix permeability. This result has validated that the fracture system is the mian fluid flow channels which is the also the assumption for the dual porosity model. In the dual porosity model, the matrix system is the main storage space and the fracture system is the main fluid flow space.

### Effect of matrix and fracture porosity

**Fig 18:**
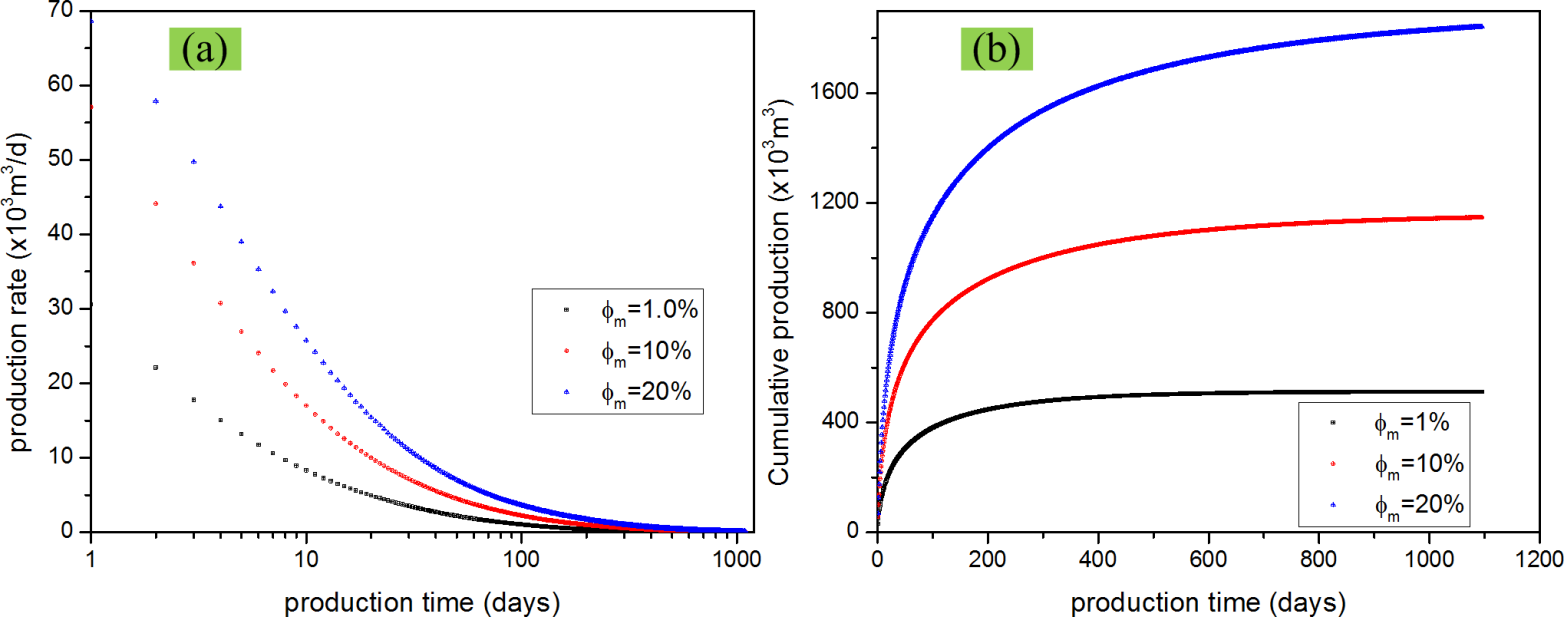
Effect of matrix porosity on gas production. (a) production rate vs. production time; (b) cumulative production vs. production time.

**Fig 19:**
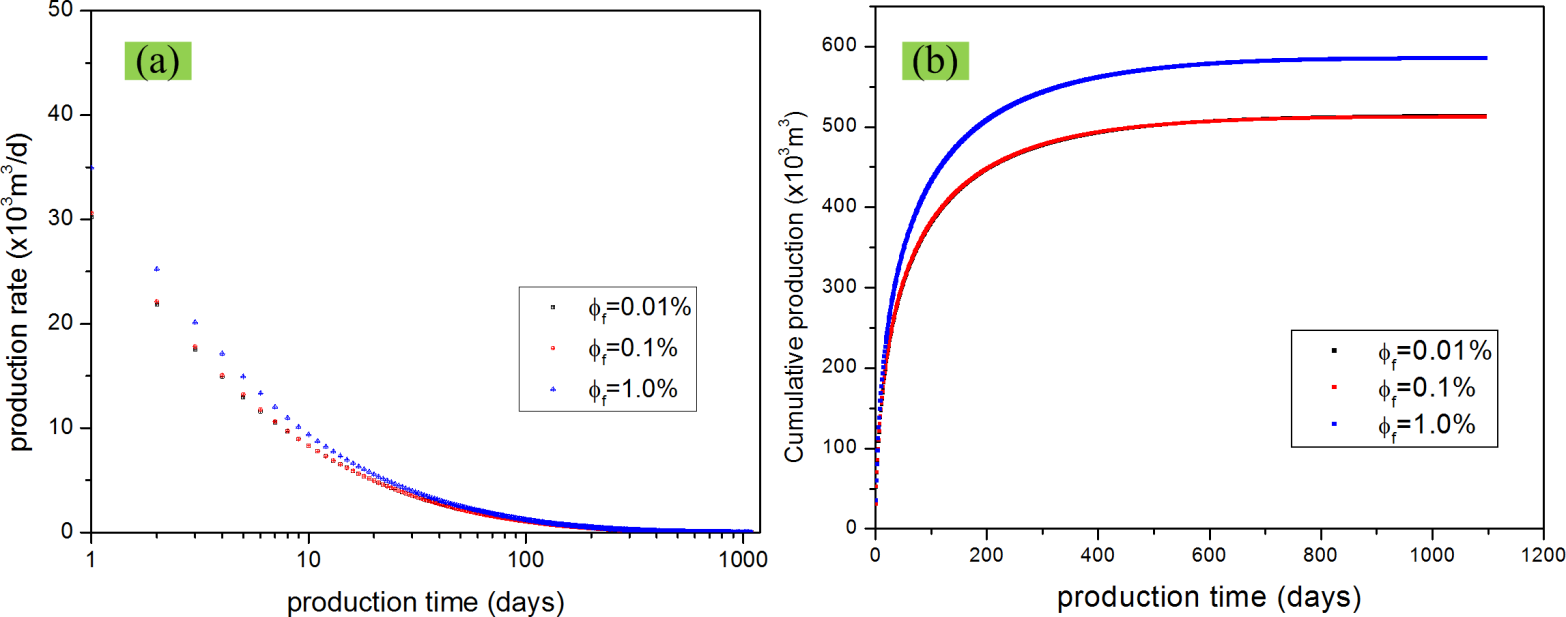
Effect of fracture porosity on gas production. (a) production rate vs. production time; (b) cumulative production vs. production time.

Porosity decides the gas amount which can be stored in the reservoir. Study on the effect of matrix and fracture porosity can help us choose target reservoir and speed history match. For studying the effect of porosity, three levels of matrix porosity and fracture porosity were considered and evaluated. We have compared the difference when we change the matrix porosity from 1% to 10% to 20% and the difference when we change the fracture porosity from 0.01% to 0.1% to 1%. From Figs 18 and 19, we can find that porosity increase leads to production increase. Also, from the comparison between Fig 18(A) and Fig 18(B), and between Fig 19(A) and Fig 19(B), it can be found that matrix porosity has a more siginificant effect on shale gas production compared with fracture porosity, which conforms to the assumption of dual porosity model.

## Conclusion

This paper presents a theoretical model and mechanism study for shale gas production. Following conclusions were obtained from this study:

A new mathematical model that considers adsorption, non-Darcy permeability change, gas viscosity change, and pore radius increase due to gas desorption was constructed;

Results analysisshowed that considering one of following scenarios: adsorption, non-Darcy permeability change, gas viscosity change, and pore radius change increases the production estimate. Among these mechanisms, adsorption and gas viscosity change have a great impact on gas production. Ignoring one of these effects decreases gas production;

Sensitivity analysis of the reservoir parameters showed that initial reservoir pressure has a great impact on gas production. Fracture permeability has a more important effect than the matrix permeability. Porosity increase leads to the increase of gas production. However, matrix porosity is more important than fracture porosity.

## Supporting Information

S1 Fig The dataset for Fig 6. (XLSX)

S2 Fig The dataset for Fig 7. (XLSX)

S3 Fig The dataset for Fig 9. (XLSX)

S4 Fig The dataset for Fig 10. (XLSX)

S5 Fig The dataset for Fig 11. (XLSX)

S6 Fig The dataset for Fig 12. (XLSX)

S7 Fig The dataset for Fig 13. (XLSX)

S8 Fig The dataset for Fig 14. (XLSX)

S9 Fig The dataset for Fig 15. (XLSX)

S10 Fig The dataset for Fig 16. (XLSX)

S11 Fig The dataset for Fig 17. (XLSX)

S12 Fig The dataset for Fig 18. (XLSX)

S13 Fig The dataset for Fig 19. (XLSX)

S1 File The dataset for Figs 6, 7, 9, 10 to 19 are included. (7Z)

## Abbreviations

α: Tangential momentum accommodation coefficient
b: Slip coefficient
b_f_: Non-darcy coefficient
b_m_: Non-darcy coefficient for the fracture system
C_m_: Gas mole concentration (mol⁄m^3^)
D_km_: Diffusion coefficient of the bedrock (m^2^/s)
$d_{CH_{4}}$: Molecular diameter of methane (m)
D_kf_: Knudsen diffusion coefficient for the fracture system (m^2^/s)
J_k_: Mmass flow caused by Knudsen diffusion (kg/(m^2^/s))
J_v_: Mass flow caused by viscous flow (kg/(m^2^/s))
k_mi_: Initial permeability of bedrock (m^2^)
k_fi_: Initial fracture permeability (m^2^)
k_f_: Apparent fracture permeability (m^2^)
k_m_: Apparent matrix permeability (m^2^)
M_g_: Gas molar mass (kg/mol)
M: Mass accumulation (kg/(m^2^.s))
p_f_: Gas pressure in the fracture (Pa)
p_m_: Gas pressure in the matrix (Pa)
p_w_: Bottomhole pressure (Pa)
p_L_: Langmuir pressure of CH_4_ (Pa)
$\overset{-}{p_{f}}$: Average pressure in the fracture (Pa)
Q: Source term (kg/(m^3^.s))
q_a_: Adsorption volume per unit mass of shale (m^3^/kg)
r_eff_: Effective pore radius (m)
r_w_: Wellbore radius (m)
r_e_: Drainage radius (m)
S_β_: Faction of pore volume occupied by phase β (%)
V_L_: Langmuir volume of CH_4_ (m^3^/kg)
V_std_: Gas mole volume under standard condition (0°C, 1atm) (m^3^/mol)
ρ: Density (kg/m^3^)
β: Fluid phase
ϕ_m_: Matrix porosity (%)
ϕ_f_: Fracture porosity (%)
ρ_s_: Density of shale core (kg/m^3^))
ρ_m_: Gas density in the bedrock (kg/m^3^))
μ_g_: Gas viscosity (Pa·s)
σ: Crossflow coefficient
